# Emotion dysregulation as mediator between mentalizing capacity and affective states: An exploratory study

**DOI:** 10.1002/pcn5.124

**Published:** 2023-07-25

**Authors:** Saeed Ghanbari, Ahmad Asgarizadeh, Elahe Vahidi, Parisa Sadat Seyed Mousavi, Maryam Omidghaemi

**Affiliations:** ^1^ Faculty of Education and Psychology Shahid Beheshti University Tehran Iran; ^2^ Family Research Institute Shahid Beheshti University Tehran Iran

**Keywords:** affective states, emotion dysregulation, mentalizing capacity

## Abstract

**Aim:**

We intended to elucidate the relationship between mentalizing capacity and affective states by investigating the mediatory role of emotion dysregulation.

**Methods:**

A sample of nonclinical Iranian adults (*N* = 445) completed a battery of online self‐report measures comprising the Positive and Negative Affect Schedule (PANAS), Mentalization Scale (MentS), and Difficulties in Emotion Regulation Scale (DERS).

**Results:**

Correlational analyses demonstrated that self‐ and other‐related mentalizing were significantly and inversely associated with emotion dysregulation, which in turn was strongly linked with positive and negative affects. Using structural equation modeling, the results delineated emotion dysregulation as a mediator between self‐ and other‐related mentalizing and affective states, predicting higher negative and lower positive affects. However, motivation to mentalize failed to predict positive affects and only contributed to lower negative affects directly.

**Conclusion:**

Mentalizing capacity was found to be indirectly linked with affective states through emotion dysregulation; hence, along with the previously substantiated association between emotion dysregulation and affective states and the partially established relationship between mentalizing capacity and affective states, we propose mentalizing incapacity to be at fault in the development of affective difficulties.

## Graphical abstract

- The capacities to mentalize oneself and others were negatively related to emotion dysregulation, which in turn was linked to lower positive and higher negative affects.
- Emotion dysregulation fully mediated the associations between self‐ and other‐focused mentalizing and negative affects, but was only a partial mediator for positive affects.
- Motivation to mentalize was directly associated with lower negative affects, but was not a significant predictor of emotion dysregulation

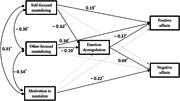

## INTRODUCTION

There is no consensus among clinicians and researchers on defining “affect.”^1^, ^2^ Occasionally, the terms “affect,” “emotion,” and “mood” are used interchangeably (e.g., mood disorders are also widely known as affective disorders). However, one could argue that these are different in several ways. For instance, emotions are transient and intertwined with physiological elements, while moods are durable subjective feelings.^3^ In the current paper, we adopt the definition of Gross,^4^ who most recently suggested “affect” to be an overarching term encompassing emotions, stress responses, and moods. Traditionally, affects or affective states are divided into two dimensions: positive affects (PA) and negative affects (NA).^5^ PA demonstrates the extent to which a person is experiencing positive feelings (e.g., interested, inspired, strong), and NA reflects the degree to which a person experiences negative feelings (e.g., distressed, nervous, ashamed). A large body of literature suggests that NA is strongly associated with psychopathology, while PA may act both as a buffer or a culprit in different disorders.^6^ Plausibly, the inability to regulate one's emotions adaptively or emotion dysregulation, which is “a pattern of emotional experience and/or expression that interferes with appropriate goal‐directed behavior,”^7^ may be a risk factor for psychopathology.^8^ Emotion dysregulation, on the other hand, is argued to have developmental roots, namely mentalizing incapacity.^9^ Mentalizing is an imaginative capacity of humans, that is, envisaging one's own and others' behaviors with reference to the underlying mental states.^10^ In the following section, we will provide theoretical and empirical evidence for the pairwise associations between affective states, emotion regulation, and mentalizing.

The theoretical basis for the association of mentalizing and enduring affective states (i.e., mood) is only implicitly posited. Also, the term “mood” is relatively rare in the publications by Fonagy and colleagues.^11^ Secure attachment and mentalizing develop jointly, with parents mirroring affects congruently and in a marked, unrealistic manner.^12^ Subject to the parents' failure in fulfilling the aforesaid prerequisites of a secure attachment relationship in early childhood, which, in turn, cultivates the ground for the development of mentalizing, individuals are likely to experience affective disarrays.^13^, ^14^ Of note, Converse et al.^15^ proposed that the quality of mood states (i.e., happiness and sadness) impact the extent to which individuals employ the theory of mind. Furthermore, an emerging body of research illustrates that mood disturbances undermine individuals' capacity to mentalize.^16^ Thus, mentalizing and affective states appear to have a bidirectional relationship. Studies examining the link between mentalizing and affective states are scarce. In a recent study, Vives et al.^17^ found that, contrary to their hypothesis and theoretical postulations, self‐focused mentalizing acted counterproductively and prolonged recovery from distress.

Emotion dysregulation might be the overlooked bridge linking mentalizing deficits and affective problems. At its core, the concept of mentalization is intertwined with emotion regulation.^9^ Marszał and Jańczak^18^ found mentalizing to partially explain emotion dysregulation in late adolescent girls, with lower levels of mentalizing corresponding to a lower ability to regulate one's emotions. Furthermore, Schultheis et al.^19^ suggested that mothers who suppressed the expression of their emotions, as well as mothers who had difficulties with emotion regulation, are likely to fall back to “pre‐mentalizing modes.”^10^ In line with the former finding, in a study of adolescent inpatients with eating disorders, self‐focused mentalizing and proclivity to suppress emotions were negatively related.^20^ As well, the relationship between mentalizing deficits and emotion dysregulation was also confirmed in different categories of psychopathology.^21^, ^22^, ^23^, ^24^, ^25^ In the most recent study, Schwarzer et al.^26^ found that other‐focused mentalizing was only associated with the adaptive regulation of anxiety, whereas self‐focused mentalizing was associated with the adaptive regulation of anger, anxiety, and sadness. Furthermore, other‐focused mentalizing was negatively related to the maladaptive regulation of anger and anxiety, while self‐focused mentalizing was negatively related to the maladaptive regulation of anger, anxiety, and sadness.

Although overlapping, emotion dysregulation is distinguishable from NA.^27^ Of note, a prospective longitudinal study suggests that emotion dysregulation is the precursor of the onset or aggravation of internalizing problems rather than vice versa^28^; thus, the above‐stated association is likely to be unidirectional.^29^ Difficulties in emotion regulation are directly associated with NA and inversely to PA.^30^, ^31^, ^32^ Moreover, findings are incongruent regarding the link between emotion regulation strategies and affective states. For instance, some studies found that emotional suppression is positively associated with PA and negatively with NA,^33^, ^34^ while others demonstrated that emotional suppression might be interpersonally beneficial when it is situationally appropriate.^35^, ^36^

### Objectives

The current study intended to elucidate the association between mentalizing capacity and affective states. In line with the above‐stated postulations and findings, we expected emotion dysregulation to mediate the relationship between mentalizing deficits and enduring affective states. Despite the poverty of empirical support, we argued that better mentalizing capacity contributes to the generation and maintenance of individuals' PA, while mentalizing incapacity predicts higher NA. Meanwhile, the link between emotion regulation and affective states has been rigorously studied, generally yielding congruous findings: emotion dysregulation is positively related to NA and negatively to PA. Moreover, since emotion regulation was suggested to be the precursor of mentalizing capacity in normal development and an outcome of sophisticated mentalizing in later life, we expected an indirect link between sophisticated mentalizing and emotion dysregulation.

## METHOD

### Participants and procedure

Participants were 445 adults (317 females) from the community with their ages ranging from 18 to 43 years (*M* = 27.53 years, *SD* = 8.71 years) recruited through convenience sampling. More than half of the second sample were college students (*N* = 249) and unmarried (*N* = 307). Table 1 demonstrates the demographic characteristics of the sample.

**Table 1:** Sociodemographic characteristics of participants.

Characteristics	Frequency	Percentage
Sex		
Female	317	71.2
Male	128	28.8
Age (years)		
18–24	189	42.5
25–34	175	39.3
35–44	57	12.8
45–54	18	4
55–65	6	1.3
Marital status		
Single	307	69
Married	138	31
Number of children		
0	344	77.3
1	41	9.2
2	41	9.2
3	14	3.1
4+	5	1.2
Discipline		
Humanities	56	12.6
Basic sciences	15	3.4
Engineering	150	33.7
Medicine	160	36
Other	64	14.4

Due to the restrictions of the COVID‐19 pandemic, the battery of the measures was created online using Porsline (https://porsline.ir), and the URL was disseminated via the most popular social media applications in Iran (i.e., Telegram, Instagram, and WhatsApp). Participants were informed about the aim of the study and the confidentiality of the gathered data and signed digital consent forms as a precondition to taking part. All the procedures in the current study were approved by the Ethics Committee of the Attachment and Interpersonal Relationships Research Center, Shahid Beheshti University.

### Measures

The Mentalization Scale (MentS)^3^ is a relatively new measure that comprises three subscales: Self‐related Mentalization (MentS‐S), with items including “I am often confused about my exact feelings”; Other‐related Mentalization (MentS‐O), with items including “I can make good predictions of other people's behavior when I know their beliefs and feelings”; and Motivation to Mentalize (MentS‐M), with items including “I do not like to waste time trying to understand in detail other people's behavior.” Each subscale consists of 10, 8, and 10 items, respectively, adding up to a total number of 28 items. MentS is scored using a point‐point Likert scale, ranging from 1 = *completely incorrect* to 5 = *completely correct*. Higher scores on the total scale and each subscale indicate a more advanced ability to mentalize. The MentS's validity was supported by significant correlations with measures of attachment, emotional intelligence, and basic personality traits.^37^ Recently, the Iranian version of the MentS was preliminarily validated in samples of nonclinical adults.^38^ In the current study, Cronbach's *α*s for the total scale, MentS‐S, MentS‐O, and MentS‐M, were 0.85, 0.79, 0.78, and 0.74, respectively.

The Positive and Negative Affect Schedule (PANAS)^5^ was used to assess the intensity of positive and negative affect. PANAS comprises two 10‐item scales measuring PA and NA. One can choose the intensity of affect on a five‐point Likert scale, ranging from 1 = *very slightly or none at all* to 5 = *extremely*. Higher scores on each scale indicate stronger affect. The respondents were asked to report the intensity of each affect in the past year. In this sample, Cronbach's alpha for NA and PA were 0.88 and 0.85, respectively.

Difficulties in Emotion Regulation Scale (DERS)^39^ is a well‐established measure that assesses emotion dysregulation, using a five‐point Likert scale, ranging from 1 = *almost never* (0%–10%) to 5 = *almost always* (91%–100%). DERS is composed of six subscales, namely Nonacceptance of Emotional Responses, Difficulties in Engaging in Goal‐Directed Behavior, Impulse Control Difficulties, Lack of Emotional Awareness, Limited Access to Emotion Regulation Strategies, and Lack of Emotional Clarity. Each of the six subscales includes six items, with a total number of 36 items. Higher total scale scores represent poorer emotion regulation. Cronbach's *α* of the total scale was 0.93 in this study.

### Data analysis

First, Pearson correlation coefficients were calculated to explore the relationships among the MentS's total score and its subscales, DERS's total score, and the NA and PA subscales of PANAS. The criteria suggested by Cohen^40^ were used to interpret correlation coefficients: negligible to very weak (*r* = 0.00–0.10), weak (*r* = 0.10–0.30), moderate (*r* = 0.30–0.50), and strong (*r* = 0.50–1.00).

**Figure 1:**
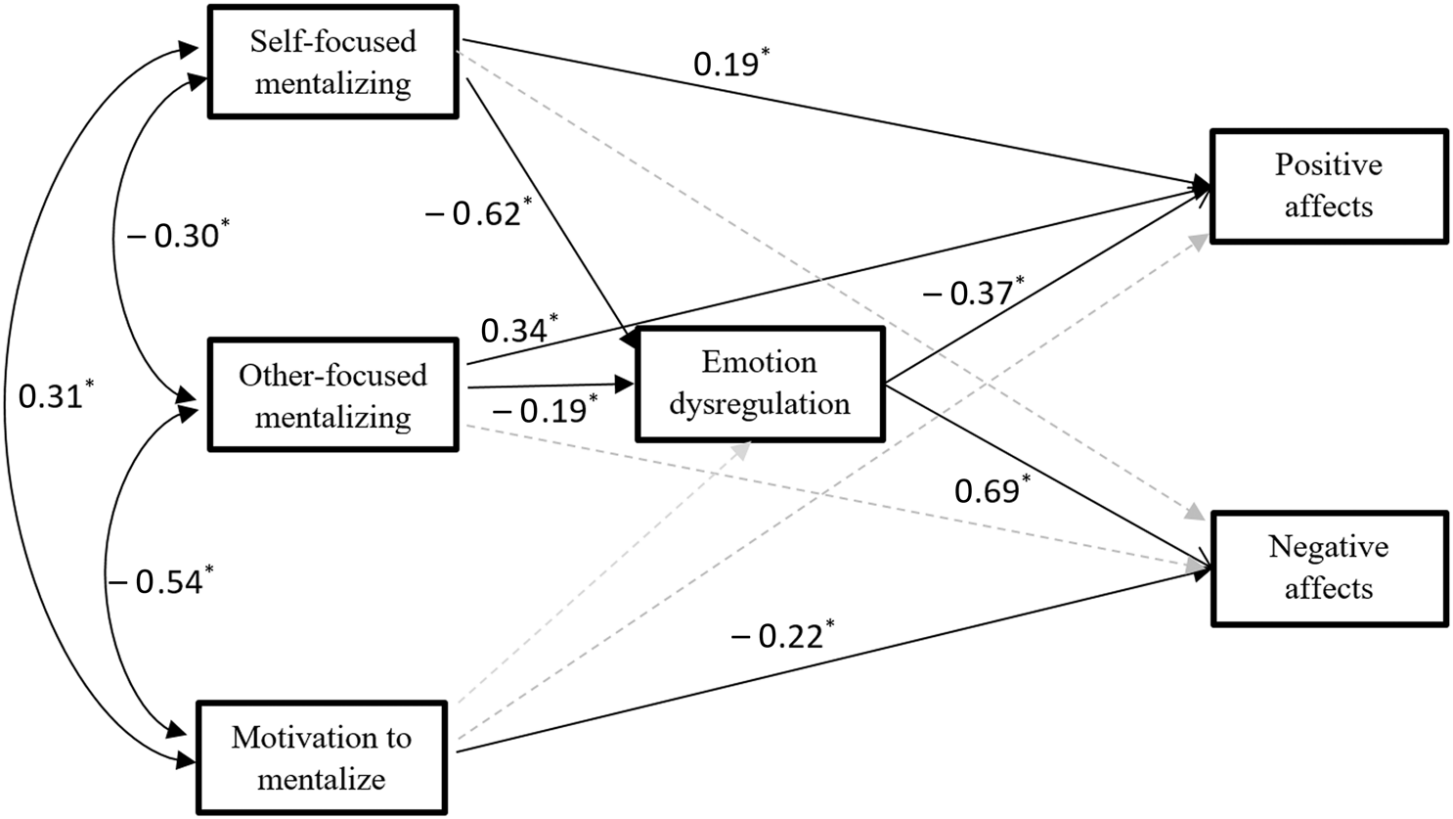
Structural model corresponding to the final model proposed by the study; pale dashed lines indicate insignificant paths. *p < 0.01.

Next, a structural equation model was used to investigate the theoretical model presented in Figure 1. The Satorra–Bentler scaled *χ* ^2^ test was used to evaluate the overall fit of the factor models. As *χ* ^2^ is sensitive to sample size, model fit was also evaluated using three additional fit indices, root mean square error of approximation (RMSEA), comparative fit index (CFI), and goodness of fit index (GFI). The following cut‐off values were used: *χ* ^2^/*df* ≤3 for an acceptable fit; CFI and GFI >0.90, and RMSEA <0.08 (acceptable fit) and <0.06 (good fit).^41^ The significance of indirect effects was tested using the bootstrapping procedure. Effects were computed for each of the 2000 bootstrapped samples. Statistical analyses were conducted using SPSS (IBM SPSS Statistics 23) and AMOS (IBM SPSS AMOS 24) software.

## RESULTS

### Zero‐order correlations

Table 2 demonstrates the descriptive statistics of the variables and the Pearson correlation coefficients between them. As Table 2 indicates, emotion dysregulation was strongly associated with MentS‐S (*r* = −0.65, *p* < 0.01), moderately associated with MentS‐O (*r* = −0.33, *p* < 0.01), weakly associated with MentS‐M (*r* = −0.21, *p* < 0.01), and strongly associated with the total score of MentS (*r* = −0.53, *p* < 0.01). There were weak to moderate associations between all of the subscales of MentS and its total score and PA. All MentS subscales and its total score were weakly to moderately associated with NA, with the exception of MentS‐M. Emotion dysregulation was moderately and negatively associated with PA (*r* = −0.36, *p* < 0.01) and strongly and positively associated with NA (*r* = 0.64, *p* < 0.01).

**Table 2:** Pearson correlations among subscales of MentS and PANAS, and DERS alongside mean and standard deviation for all variables.

	1	2	3	4	5	6	Mean (SD)
1. MentS‐S	1						27.77 (6.07)
2. MentS‐O	0.31**	1					39.72 (5.06)
3. MentS‐M	0.32**	0.55**	1				39.33 (5.48)
4. MentS‐Total	0.73**	0.78**	0.80**	1			106.82 (12.73)
5. DERS	−0.65**	−0.33**	−0.21**	−0.52**	1		85.29 (23.46)
6. PA	0.15**	0.39**	0.11*	0.28**	−0.36**	1	35.39 (6.55)
7. NA	−0.39**	−0.10*	0.07	−0.19**	0.64**	−0.22**	27.29 (7.72)

Abbreviations: DERS, Difficulties in Emotion Regulation Scale; MentS‐S, Mentalization Scale – Self subscale; MentS‐O, Mentalization Scale – Others subscale; MentS‐M, Mentalization Scale –Motivation subscale; MentS‐Total, total score of Mentalization Scale; NA, negative affects; PA, positive affects; PANAS, Positive and Negative Affect Schedule.

** *p* < .01

* *p* < .05.

### Structural equation modeling

The proposed model (Figure 1) provided a good fit for the data. However, in this model, several paths, including the paths from MentS‐M to difficulties in emotion regulation and PA, the path from MentS‐S to NA, and the path from MentS‐O to NA, were insignificant. These paths were removed, which led to the final model with a good fit *χ* ^2^ (5) = 12.14, *p* = 0.03, RMSEA = 0.07, 90% CI =  [0.02–0.09], CFI = 0.99, GFI = 0.99). The standardized regression coefficients are presented in Figure 1. The explained variance by the predictors in the model for difficulties in emotion regulation, PA, and NA were 0.44, 0.24, and 0.45, respectively. Results of the bootstrapping procedure regarding the standardized indirect and total effects are presented in Table 3.

**Table 3:** Indirect effects for the hypothesized mediations.

		Bootstrapping bias‐corrected 95% CI	
Indirect effect	*Β*	Lower	Upper
MentS‐S → DERS → PA	0.23*	0.17	0.29
MentS‐S → DERS → NA	−0.42*	−0.47	−0.37
MentS‐O → DERS → PA	0.07*	0.05	0.11
MentS‐O → DERS → NA	−0.13*	−0.18	−0.08

Abbreviations: DERS, Difficulties in Emotion Regulation Scale; MentS‐S, Mentalization Scale – Self subscale; MentS‐O, Mentalization Scale – Others subscale; NA, negative affects; PA, positive affects.

* *p* < 0.01.

## DISCUSSION

We examined the role of mentalizing capacity in the emergence of PA and NA, as mediated by emotion dysregulation. The capacities to mentalize oneself and others were negatively related to emotion dysregulation, which in turn was linked to lower PA and higher NA. Moreover, both self‐focused and other‐focused mentalizing directly contributed to PA, while the same pattern did not emerge for NA. Nonetheless, emotion dysregulation did not mediate the relationship between motivation to mentalize and positive and negative affects.

Our finding regarding the inverse association between self‐ and other‐focused mentalizing and emotion dysregulation, with stronger relationships for self‐focused mentalizing, was in line with previous studies.^20^, ^26^ One possible explanation inheres in the measure we administered. DERS consists of six subscales, at least three of which directly echo the deficiency in the capacity to mentalize emotions in the self (i.e., awareness, clarity, and nonacceptance), while none of them reasonably relate to mentalizing others. We also found emotion dysregulation positively related to NA and conversely related to PA. This association was stronger for NA, which is congruent with previous findings.^31^ As explained earlier, difficulties in emotion regulation may lead to intensified and long‐lasting NA, which, in turn, can contribute to the development of various forms of psychopathology.^42^ Alternatively, the link between emotion dysregulation and NA could be considered a bidirectional one. When individuals find themselves in negative affective states, they are inclined to resolve the situation using emotion regulation strategies, particularly maladaptive ones.^32^, ^43^, ^44^

Additionally, motivation to mentalize was not a significant factor in predicting emotion dysregulation. MentS‐M represents a curiosity in the mental states of the self and others, which is mainly called an inquisitive stance.^10^ This curiosity is one of the key characteristics in fostering emotion regulation and mentalizing.^45^, ^46^ However, our findings suggest that an inquisitive stance, or in other words, motivation to mentalize, does not predict the regulation of emotions. Conceptually, this finding corresponds to the multi‐dimensional nature of mentalizing. For instance, in individuals who hypermentalize frequently, the motivation to attribute mental states to self and others exists, but it is related to emotion dysregulation rather than emotion regulation.^25^

By enabling the individual to reflect on his and others' mental states, mentalizing can preserve his psychic equilibrium, thus protecting him from falling into a cascade of undesirable responses.^11^ In our viewpoint, incapacity to mentalize or momentary loss of this capacity may lead to the employment of situationally maladaptive strategies, which could further intensify NA and abate PA. If reckoning the link between affective states and emotion dysregulation is a bidirectional one, an incapacity to mentalize could trigger a vicious cycle, exacerbating individuals' affective dysphoria and emotion dysregulation in a cascade.

The findings of this study could be prudently translated into the clinical realm. Current attempts in the classification of psychopathology, particularly personality pathology, are oriented toward dimensional approaches and transdiagnostic aspects.^47^ For instance, compared to the categorical classifications, the alternative model for personality disorders^48^ has demonstrated superior diagnostic utility and perceived clinical utility.^49^ The latest manuals for mentalization‐based treatment have, in fact, moved towards incorporating this alternative dimensional model.^50^, ^51^ In this model, NA is at the forefront of the five maladaptive traits, the existing practice recommendations for addressing which encourage a particular focus on cultivating mentalizing capacity and emotion regulation.^52^, ^53^ On the other hand, attending to PA in mentalization‐based treatment is proposed to be an effective tool, even more so than NA.^50^, ^54^ Hence, the findings of this study empirically support the recent suggestions for clinical practice.

### Limitations and future directions

This study extended the empirical findings on the association between mentalizing capacity, emotion dysregulation, and affective states. Nonetheless, when interpreting our findings, a number of methodological limitations should be noted. Our findings and interpretations are only based on self‐report instruments, the use of which may be imprudent on several accounts (e.g., participants' biases in reporting). Furthermore, researchers have cautioned against the use of self‐report measures for assessing mentalizing capacity.^55^ It is safe to say that, rather than genuine mentalizing, we measured individuals' self‐rated mentalizing abilities. As a result, we advise future studies to measure mentalizing using multimethod approaches or methods that minimize biases.^56^, ^57^ Moreover, structural equation modeling requires strong assumptions (e.g., linearity for included variables), which may not be verified in this study. Also, this was a cross‐sectional exploratory study that did not control for the confounding factors and used goodness‐of‐fit evaluations; thus, causal inferences are not allowed. Hence, longitudinal studies are needed to establish the role of mentalizing incapacity and emotion dysregulation in increasing NA and decreasing PA. The use of convenience sampling may have also led to selection bias.^58^ Lastly, by virtue of the context‐dependency of mentalizing capacity,^59^ participants' mental states at the time of assessment could compromise the validity of our findings.

## AUTHOR CONTRIBUTIONS

*Conceptualization*: Saeed Ghanbari, Parisa Sadat Seyed Mousavi, Elahe Vahidi. *Methodology*: Elahe Vahidi, Maryam Omidghaemi. *Formal analysis and investigation*: Elahe Vahidi. *Writing—original draft preparation*: Ahmad Asgarizadeh, Elahe Vahidi. *Writing—review and editing*: Ahmad Asgarizadeh, Elahe Vahidi, Saeed Ghanbari, Parisa Sadat Seyed Mousavi. *Supervision*: Saeed Ghanbari, Parisa Sadat Seyed Mousavi.

## CONFLICT OF INTEREST STATEMENT

The authors declare no conflicts of interest.

## ETHICS APPROVAL STATEMENT

All procedures performed in the current study involving human participants were in accordance with the ethical standards of the Attachment and Interpersonal Relationships Research Center at Shahid Beheshti University and with the 1964 Helsinki Declaration and its later amendments or comparable ethical standards.

## PATIENT CONSENT STATEMENT

Participants were informed about the aim of the study and the confidentiality of the gathered data and signed digital consent forms as a precondition to taking part.

## CLINICAL TRIAL REGISTRATION

N/A

## Data Availability

The data that support the findings of this study are available from the corresponding author upon reasonable request.
